# Similarity-based Regularized Latent Feature Model for Link Prediction in Bipartite Networks

**DOI:** 10.1038/s41598-017-17157-9

**Published:** 2017-12-05

**Authors:** Wenjun Wang, Xue Chen, Pengfei Jiao, Di Jin

**Affiliations:** 1 School of Computer Science and Technology, Tianjin University, Tianjin, 300354 China; 2 Tianjin Engineering Center of SmartSafety and Bigdata Technology, Tianjin University, Tianjin, 300354 China; 3 Tianjin Key Laboratory of Advanced Networking (TANK), Tianjin Key Laboratory, Tianjin, 300354 China

## Abstract

Link prediction is an attractive research topic in the field of data mining and has significant applications in improving performance of recommendation system and exploring evolving mechanisms of the complex networks. A variety of complex systems in real world should be abstractly represented as bipartite networks, in which there are two types of nodes and no links connect nodes of the same type. In this paper, we propose a framework for link prediction in bipartite networks by combining the similarity based structure and the latent feature model from a new perspective. The framework is called Similarity Regularized Nonnegative Matrix Factorization (SRNMF), which explicitly takes the local characteristics into consideration and encodes the geometrical information of the networks by constructing a similarity based matrix. We also develop an iterative scheme to solve the objective function based on gradient descent. Extensive experiments on a variety of real world bipartite networks show that the proposed framework of link prediction has a more competitive, preferable and stable performance in comparison with the state-of-art methods.

## Introduction

With the rapid development of the Internet, the computational analysis of social networks has grown to be a salient issue. One of the important topics in network analysis is to explore the structures and functions of complex networks, and a considerable amount of attention has been devoted to the issue of link prediction. The process of network formation associated with the method capability of predict missing links^1^. Link prediction aims to estimate the likelihood of the existence of a link between two nodes from available network information, such as the observed links and the attributes of nodes^2,3^. For instance, discovery of underground groups of terrorists or criminals can be viewed as predicting missing links in social security networks. The nature of link prediction can be divided into two categories. One is the prediction of existing yet unknown links, such as protein-protein interaction networks and metabolic networks, the other is the prediction of links that may appear in future evolving networks, like online social networks. For the former, the discovery of links among nodes requires costly experiments because of blindly checking all possible links. Making predictions based on the links already known and focusing on those links which are most likely to exist may sharply reduce costs^4^. For the latter, in recommendation systems^5,6^, link prediction can be utilized to discover the links that are most likely to emerge in the future.

Most of link prediction approaches have been proposed on monopartite networks. The most widely used methods are the similarity-based algorithms^2,7^ and the supervised learning algorithms^8^. Besides the above prediction algorithms, some novel algorithms based on maximum-likelihood^7,9,10^ have been proposed. For the hierarchical structure of networks, Clauset *et al.* ^9^ proposed a model to infer hierarchical structure from network and applied it to solve the link prediction problem. GuimerÃ *et al.* ^10^ developed a Stochastic Block Model to capture the community structure and to estimate the probability that two nodes are connected. Pan *et al.* ^7^ proposed an algorithmic framework of probability by denoting a predefined structural Hamiltonian based on the network organizing, and predicted each non-observed link by computing the conditional probability of adding the link to the observed network. In particular, their work was able to identify both missing and spurious interactions in noisy network observations.

However, in the real world, a variety of complex systems in various fields can be modeled as bipartite networks^11^. There are two disjoint sets of nodes in bipartite networks and links may occur only if the nodes belong to different sets. Taking a metabolic network as an example, chemical substances and chemical reactions are the two different types of nodes and there are only links between reactions and substances. In an online purchasing network, the two types of nodes are users and products respectively, and the links represent the purchase relations between them. Also of note, link prediction in bipartite networks is important for providing priceless information to improve e-commerce or to accelerate biological function research.

Because of bipartite networks’ particularity, most of the existing methods for link prediction are not suitable for it. To address the problem, some approaches have been developed and we mainly classify them into three categories, projection based methods, topological structure based methods, and latent feature model.

Projection based methods project the bipartite network into two monopartite networks and exploit one or both monopartite layers obtained from a bipartite network to predict new links^12,13^. These methods infer the presence of links between any two nodes, belonging to the same layer, as long as sharing at least one neighbor. It is obvious that these methods lose the original topological structure information of the bipartite network^14^.

The second and most widely used methods are based on the topological structure in bipartite networks. The preferential attachment (PA)^15,16^ algorithm only considers the node degrees information and thus can be directly applied to link prediction in bipartite networks. It achieves higher accuracy than various algebraic (e.g. matrix factorization) methods in many real world bipartite networks^17^. Based on the formal definitions of similarity-based indices in monopartite networks, Cannistraci *et al.* ^18^ proposed related variations similarity indices in bipartite networks, including Common Neighbors (CN), Jaccard’s index (JC), Adamic Adar (AA) and allocation of resources (RA). Recently, a shift in perspective is from nodes to community links has been proposed^18–20^. The number of common neighbors and the number of local community links (links connecting common neighbors) have been taken into account by Cannistraci *et al.* ^18,20^, and they proposed a series of similarity indices to enhance the performance of link prediction in monopartite networks^20^ and bipartite networks^18^, including Cannistraci-Alanis-Ravasi (CAR), Cannistraci-Jaccard (CJC), Cannistraci preferential attachment (CPA), Cannistraci-Adamic-Adar (CAA), Cannistraci resource allocation (CRA). However, topological structure based methods consider only partial network characteristics.

Latent feature model always assumes that each node of the network is associated with a latent feature vector, and then the probability of a link is determined by the interactions among such latent features^21,22^. In details, in a network with *n* nodes, latent feature model represents each node *i* by a low-dimensional feature vector, which is a point in a latent feature space, and two nodes are more likely to be linked if they have similar latent features. From another perspective, the similarity matrix of the network can be approximated to the product of two lower ranked matrixes, which are basis matrix and coefficients matrix respectively. If we restrict the elements of the two matrixes to be nonnegative, the solution can be obtained by the algorithm of Nonnegative Matrix Factorization (NMF), which has been used to analyze complex networks successfully. Compared with other methods, latent feature model can learn expressive representations from network structures. However, the intrinsic geometrical and discriminating structure of the data space cannot be revealed, as discussed by Cai D, He X, *et al.* ^23^.

Inspired by the idea of manifold learning^24,25^ and graph regularized Nonnegative Matrix Factorization^23^, in this paper we propose an algorithm framework for link prediction in bipartite networks by combining the topological structure and the latent feature model from a new perspective. The framework is called Similarity-based Regularized Nonnegative Matrix Factorization (SRNMF), which explicitly considers the local similarity of the networks. We encode the geometrical information of the nodes space by constructing a similarity-based matrix. By incorporating the topological similarity structure, a new matrix factorization objective function is designed to find a parts-based representation space in which two nodes are both sufficiently close to each other in the space and in the similarity-based matrix. We also develop an iterative algorithm to optimize the objective function based on gradient descent. In the experiments, the proposed framework demonstrates a more competitive, preferable and stable performance on a variety of real-world bipartite networks compared with state-of-the-art methods.

## Results

Considering an undirected bipartite network *G*(*V*, *W*, *E*), in which *V* and *W* are the two sets of disjoint nodes and *E* is the set of links. Given one network, we denote its adjacency matrix $A\in {\{\mathrm{0,}1\}}^{N\times M(n=|V|,m=|W|)}$, where the element *A*_*ij*_ = 1 if nodes *v*_*i*_ and *w*_*j*_ are connected and *A*_*ij*_ = 0 otherwise. To test the algorithm’s accuracy, the observed links *E* is randomly divided into two parts. The training set *E* ^*T*^ is treated as known information, and the probe set *E* ^*P*^ is used for testing the performance of methods for link prediction. It is clear that *E* ^*T*^∪*E* ^*P*^ = *E* and *E* ^*T*^∩*E* ^*P*^ = ∅. The corresponding adjacency matrix of the training set and the probe set can be represented by *A* ^*T*^ and *A* ^*P*^ respectively, also of note, they have the same size as A.

### A framework of similarity-based regularized latent feature model

**Figure 1:**
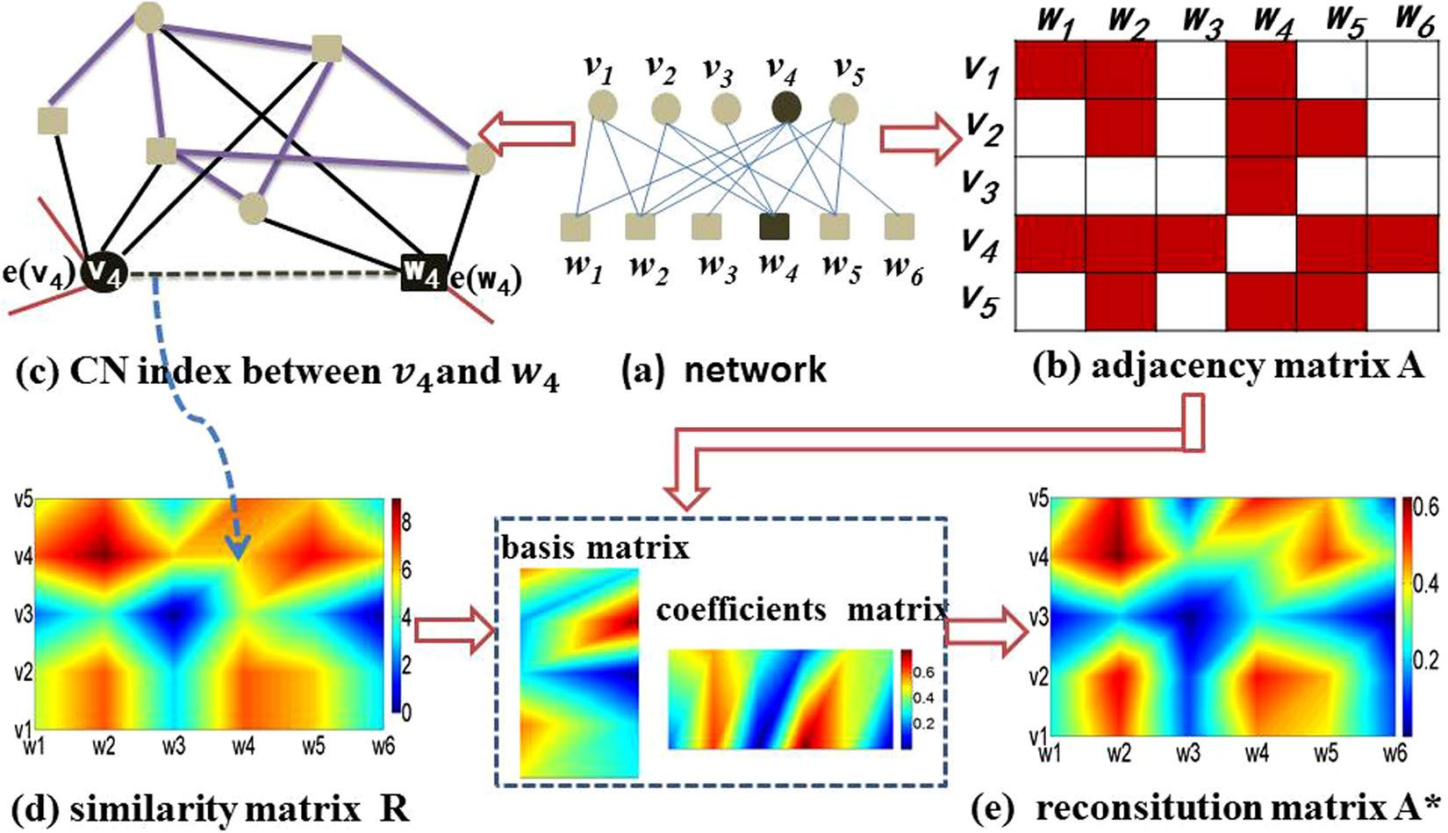
Framework of similarity-base regularized nonnegative matrix factorization. (**a**) An example of bipartite network. (**b**) Adjacency matrix of bipartite network. (**c**) CN measure between *v*_4_ and *w*_4_. (**d**) The CN similarity matrix of bipartite network. (**e**) Reconsitution adjacency matrix *A* ^*^.

In this paper, we propose a framework for link prediction in bipartite networks by combining the topological structure and the latent feature model from a new perspective. The framework exploits the intrinsic similarity structure of the nodes and which is incorporated as an additional regularization term. By preserving the similarity structure, our framework has more discriminating power than the latent feature model. The framework is shown in Fig. 1. In detail, for each pair nodes, *i* ∈ *V*, *j* ∈ *W*, we assign a score, *S*_*ij*_, according to a given similarity measure. Higher score means higher similarity between *i* and *j*, and vice versa. Figure 1(c) gives the example of calculating CN measure in bipartite network. The CN measure between node *v*_4_ and *w*_4_ is *CNs* = 6, *CNs* counts the number of neighbours touched by the quadrangles that pass through the nodes *v*_4_ and *w*_4_.

Combining this similarity-based regularizer with the latent feature model, we can get the following objective function *O*

1 $$O=\sum _{i=1}^{n}\sum _{j=1}^{m}{\boldsymbol{l}}({A}_{ij},{A}_{ij}^{\ast }(\theta ))+\gamma \sum _{i=1}^{n}\sum _{j=1}^{m}{\boldsymbol{l}}({A}_{ij},{A}_{ij}^{\ast }(\theta )){S}_{ij}+{\rm{\Omega }}(\theta )$$

where *θ* is a parameter vector, ${\boldsymbol{l}}(.,.)$ is a loss function, Ω(·) is a regularization term that prevents overfitting, ${A}_{ij}^{\ast }(\theta )$ is the model’s predicted score for (*i*, *j*), and the regularization parameter *γ* controls the smoothness of the new representation.

Such a loss function can be constructed by using some measures of distance between two matrices *A* and *A* ^*^. For example, the cost function with the square of the Euclidean distance can be written as

2 $${\boldsymbol{l}}({A}_{ij},{A}_{ij}^{\ast }(\theta ))=||A-{A}^{\ast }|{|}_{F}^{2}=\sum _{ij}{({A}_{ij}-{A}_{ij}^{\ast })}^{2}$$

The cost function with Kullback-Leibler divergence can be written as

3 $${\boldsymbol{l}}({A}_{ij},{A}_{ij}^{\ast }(\theta ))=D(A||{A}^{\ast })=\sum _{ij}{A}_{ij}\,\mathrm{log}\,\frac{{A}_{ij}}{{A}_{ij}^{\ast }}-{A}_{ij}+{A}_{ij}^{\ast }$$

Specifically, in this paper we propose the objective function of the framework in view of the nonnegative matrix factorization and cost function with the square of the Euclidean distance. Therefore, we transform the solution of *A* ^*^ into solving optimal problem of NMF. By optimizing the objective function, we can obtain the basis matrix *X* and coefficients matrix *Y*. Finally, we get the reconstructed adjacency matrix *A* ^*^ = *XY*. Details can be illustrated in section Methods.

### Evaluation Metrics

To quantify the prediction accuracy, we use the precision^26^ and AUC (area under the receiver operating characteristic curve)^27^ to measure the quality of the prediction results in this paper. The precision represents the ratio of correct edges recovered out of the top *L* edges in the candidate list generated by each link predictor. This operation is repeated 100 times for each network and the mean for each method is reported. Given the ranking of the unobserved links, precision is defined as

4 $$Precision=\frac{{L}_{r}}{L}$$

where *L* is the number of the predicted links, i.e. the number of links in *A* ^*P*^, and *L*_*r*_ is the number of correctly predicted links based on the methods. Clearly, large value of the precision means better prediction of the method.

AUC metric can be interpreted as the probability that a randomly chosen link in *E* ^*P*^ (i.e., a missing link that indeed exists but is not observed yet) is ranked higher than a randomly chosen link in *U*-*E* (i.e., a nonexistent link)^7^, here *U* is the set of all possible node pairs in a network. Among *n* independent comparisons, if there are *n*′ occurrences of missing links having a higher score and *n*″ occurrences of missing links and nonexistent link having the same score, we could compute the AUC as:

5 $$AUC=\frac{n^{\prime} +0.5n^{\prime\prime} }{n}$$

In general, a larger AUC value indicates higher performance. Hence, the AUC value of the perfect result is 1.0, whereas the value of AUC gennerated by a random predictor is 0.5.

### Datasets and Baseline Algorithms

To test the performance of our proposed framework, we consider the following eight real-world networks (i) G-protein coupled receptors (GPC Receptors)^28^: The biological network of drugs binding GPC receptors. (ii)Ion channels^28^: The biological network of drugs binding ion channel proteins. (iii) Enzymes^28^: The biological network of drugs binding enzyme proteins. (iv) Southern Women^29^ (referred here as “SW”): The social relations network of women and events. (v) Malaria^30,31^: The genetic network consisting of genetic sequences from the malaria parasite plasmodium falciparum. (vi) Drug-target^32^: The chemical network of drug-target interaction. (vii) Country-organization^33^: The network of organization most related to the country. (viii) Na-net^34^: The Air transportation network, with city identifiers and coordinates. (ix) MovieLens (http://www.grouplens.org): The bipartite networks of users and movies. In the dataset each user gives any movie a rating from 1–5. If the rating is not less than 3, then we can draw a link between the user and the movie. The detailed information about these datasets is described in Table 1.

**Table 1:** Statistics of the networks studied in this paper.

network	|*V*|	|*W*|	|*E*|	LD	AD	LAD	RAD
GPC	95	223	635	0.0300	2.00	6.68	2.85
Enzymes	664	445	2926	0.0099	2.64	4.41	6.58
Ionchannel	210	204	1476	0.0345	3.57	7.03	7.24
Malaria	297	806	2965	0.0124	2.69	9.98	3.68
Drug-target	200	150	454	0.0151	1.30	2.27	3.03
Southern Women	18	14	89	0.3532	2.78	4.94	6.36
Country-organization	144	151	12170	0.5597	41.25	84.51	80.60
Na-net	940	940	12170	0.0078	3.67	7.33	7.33
Movielens	1682	943	85250	0.0537	32.48	50.68	90.40

Where, |*V*|, |*W*| denote the number of two types of nodes respectively. |*E*|, LD, AD, LAD, and RAD are the number of edges, the link density, the average degree, the left average degree, the right average degree.

For comparison, we introduce some benchmark methods, which are defined in the following examples below. The first ten methods are based on topological structure. NMF, the eleventh method, directly predicts the links on bipartite adjacency matrix, and learns latent features from network. The last six methods are projection-based methods.

- Common Neighbors (CN)^18^, which denotes the similarity measure of two different types of nodes *x* and *y* as
  6 $${s}_{xy}^{CN}=|(N(x)\cap N(N(y)))\cup (N(y)\cap N(N(x)))|$$
  where *N*(*x*) and *N*(*y*) indicate the first-order neighbours, and *N*(*N*(*x*)) and *N*(*N*(*y*)) represent the second-order neighbours of the nodes *x* and *y*, respectively. CN measure in bipartite networks counts the neighbours touched by the quadrangle that passes through the nodes *x* and *y* ^18^. For instance, in Fig. 1(c) the CN index of two nodes *v*_4_ and *w*_4_ equals to 6.
- Jaccard Coefficient (JC)^18^ is denoted as
  7 $${s}_{xy}^{JC}=\frac{{s}_{xy}^{CN}}{|N(x)\cup N(y)|}$$
- Adamic Adar (AA)^18^ is denoted as
  8 $${s}_{xy}^{AA}={\sum }_{z\in (N(x)\cap N(N(y)))\cup (N(y)\cap N(N(x)))}\frac{1}{{\mathrm{log}}_{{\rm{2}}}|N(z)|}$$
  which considers the information about the degree of the common neighbors of two different types of nodes *x* and *y*, and assigns the low-connected neighbors with more weight.
- Resources Allocation (RA)^18^ is denoted as
  9 $${s}_{xy}^{RA}={\sum }_{z\in (N(x)\cap N(N(y)))\cup (N(y)\cap N(N(x)))}\frac{1}{|N(z)|}$$
  the RA index assigns the different weight to the common neighbors of the two different types of nodes.
- Preferential attachment (PA)^16^ is denoted as
  10 $${s}_{xy}^{PA}=|N(x)|\cdot |N(y)|$$
- Cannistraci-Alanis-Ravasi (CAR)^18^ is denoted as
  11 $${s}_{xy}^{CAR}={s}_{xy}^{CN}\cdot {s}_{xy}^{LCL}$$
  where ${s}^{LCL}$ counts the links (purple colour) between the common neighbors. In Fig. 1(c), the *LCL* index of two nodes *x* and *y* equals to 7.
- Cannistraci-Jaccard (CJC)^18^ is denoted as
  12 $${s}_{xy}^{CJC}=\frac{{s}_{xy}^{CAR}}{|N(x)\cup N(y)|}$$
- Cannistraci-Adamic-Adar (CAA)^18^ is denoted as
  13 $${s}_{xy}^{CAA}={\sum }_{z\in (N(x)\cap N(N(y)))\cup (N(y)\cap N(N(x))))}\frac{|\gamma (z)|}{{\mathrm{log}}_{2}|N(z)|}$$
  where |*γ*(*z*)| is the local community degree of *z* and corresponds to *LCL* that originates from *z*;
- Cannistraci resource allocation (CRA)^18^ is denoted as
  14 $${s}_{xy}^{CRA}={\sum }_{z\in (N(x)\cap N(N(y)))\cup (N(y)\cap N(N(x)))}\frac{|\gamma (z)|}{|N(z)|}$$
- Cannistraci preferential attachment (CPA)^18^ is denoted as
  15 $${s}_{xy}^{CPA}=e(x)\cdot e(y)+e(x)\cdot {s}_{xy}^{CAR}+e(y)\cdot {s}_{xy}^{CAR}+{({s}_{xy}^{CAR})}^{2}$$
  where *e*(*x*) is the external degree of node *x*, and is presented in Fig. 1(c) (red edges);
- Nonnegative Matrix Factorization (NMF)^35^, which learns the representation parts of the original network by approximating the adjacency matrix into the product of two low-rank matrices, and has been developed to predict links with low-rank approximation.
- Jaccard (Jac)^36^ measures similarity between the same type of nodes *x*_1_ and *x*_2_. Jaccard uses the size of the intersection divided by the size of the union of it.
- Euclidean (Euc)^36^ measures similarity between the same type of nodes *x*_1_ and *x*_2_ by the concept of Euclidean distance.
- Cosine (Cos)^36^ is based on the Cosine similarity between the same type of nodes *x*_1_ and *x*_2_.
- Pearson (Pea)^36^ is based on the well-known Pearson correlation coefficient.
- Bipartite projection via Random-walk (BPR)^36^ defines a new similarity measure that utilizes a practical procedure to extract monopartite graphs without making a priori assumptions about underlying distributions.
- Network-based inference (NBI)^13^ computes the similarity between nodes in a projected network. NBI is based on resource allocation, and also takes the network structure into account.

### Experiment results

In this section, we compare our SRNMF method with seventeen widely applied link prediction algorithms in bipartite network, consist of topological structure based methods (including CN, JC, AA, RA, CAR, CJC, CPA, CAA and CRA), projection-based methods (including Jac, Euc, Cos, Pea, BPR, NBI) and NMF methods. See “Baseline Algorithms” for details. In our experiments, we set $\gamma =\frac{1}{2}$, *λ* = 2. The prediction accuracy measured by precision and AUC is shown in Tables 2 and 3 respectively. For each of the nine networks, the training set contains 90% of the links, and the remaining 10% of links constitute the probe set. Among all the comparable indices the overall prediction performance of SRNMF outperforms significantly.

**Table 2:** The prediction accuracy measured by precision on the 9 real networks.

Precision	GPC	Enzymes	Ionchannel	Malaria	Drug-target	SW	Na-net	Movielens	Country- organization
CN	0.31\19	0.37\19	0.23\21	0.19\18	0.61\16	0.14\21	0.29\16	0.14\19	0.87\15
RA	0.33\13	0.30\21	0.26\20	0.22\16	0.69\11	0.18\16	0.29\16	0.10\23	0.89\13
AA	0.32\17	0.29\22	0.21\22	0.22\16	0.64\14	0.17\18	0.30\13	0.13\20	0.87\15
JC	0.01\25	0.03\23	0.02\25	0.25\12	0.38\23	0.02\27	0.00\24	0.00\26	0.60\25
PA	0.08\23	0.02\25	0.04\24	0.02\24	0.31\24	0.12\23	0.22\21	0.15\18	0.87\15
CAR	0.33\13	0.52\13	0.48\15	0.19\18	0.60\18	0.19\12	0.30\13	0.18\11	0.87\15
CRA	0.37\11	0.65\12	0.56\12	0.25\12	0.63\15	0.21\7	0.33\3	0.18\11	0.88\14
CAA	0.32\17	0.50\16	0.53\13	0.19\18	0.59\19	0.12\23	0.27\20	0.18\11	0.87\15
CJC	0.36\12	0.51\15	0.53\13	0.23\15	0.61\16	0.19\12	0.29\16	0.18\11	0.87\15
CPA	0.33\13	0.52\13	0.48\15	0.19\18	0.59\19	0.18\16	0.30\13	0.18\11	0.87\15
NMF	0.01\25	0.00\26	0.01\26	0.00\27	0.02\26	0.03\26	0.00\24	0.00\26	0.00\27
Cos	0.20\22	0.33\20	0.35\19	0.14\22	0.49\22	0.16\19	0.16\23	0.12\22	0.66\23
Euc	0.04\24	0.02\25	0.05\23	0.01\25	0.15\25	0.12\23	0.00\24	0.05\25	0.62\24
Jac	0.21\21	0.42\18	0.40\18	0.13\23	0.52\21	0.20\9	0.18\22	0.13\20	0.74\22
Pea	0.01\25	0.00\26	0.00\27	0.01\25	0.00\27	0.14\21	0.00\24	0.07\24	0.58\26
BPR	0.27\20	0.50\16	0.44\17	0.25\12	0.68\13	0.16\19	0.29\16	0.18\11	0.93\4
NBI	0.33\13	0.68\10	0.59\11	0.26\4	0.69\11	0.19\12	0.32\12	0.18\11	0.93\4
SRNMF-CN	0.41\10	0.69\1	0.69\1	0.26\4	0.74\1	0.20\9	0.36\1	0.19\1	0.94\1
SRNMF-RA	0.42\3	0.69\1	0.68\9	0.26\4	0.74\1	0.22\2	0.33\3	0.19\1	0.92\10
SRNMF-AA	0.43\1	0.69\1	0.69\1	0.26\4	0.74\1	0.22\2	0.33\3	0.19\1	0.92\10
SRNMF-JC	0.43\1	0.69\1	0.69\1	0.27\1	0.74\1	0.23\1	0.35\2	0.19\1	0.93\4
SRNMF-PA	0.42\3	0.69\1	0.69\1	0.26\4	0.72\10	0.22\2	0.33\3	0.19\1	0.91\12
SRNMF-CAR	0.42\3	0.68\10	0.69\1	0.26\4	0.73\7	0.22\2	0.33\3	0.19\1	0.94\1
SRNMF-CRA	0.42\3	0.69\1	0.68\9	0.26\4	0.73\7	0.19\12	0.33\3	0.19\1	0.93\4
SRNMF-CAA	0.42\3	0.69\1	0.69\1	0.27\1	0.74\1	0.20\9	0.33\3	0.19\1	0.93\4
SRNMF-CJC	0.42\3	0.69\1	0.69\1	0.26\4	0.73\7	0.22\2	0.33\3	0.19\1	0.94\1
SRNMF-CPA	0.42\3	0.69\1	0.69\1	0.27\1	0.74\1	0.21\7	0.33\3	0.19\1	0.93\4

We compare our SRNMF method with seventeen well-known methods presented in baseline algorithms. For each real network, 10% of its links will be randomly selected to constitute the probe set, and the rest of the links constitute the training set. Prediction accuracy is measured by precision. The numbers behind the slash denote the ranking.

**Table 3:** The prediction accuracy measured by AUC on the 9 real networks.

AUC	GPC	Enzymes	Ionchannel	Malaria	Drug-target	SW	Na-net	Movielens	Country- organization
CN	0.81\20	0.85\20	0.91\15	0.90\18	0.92\11	0.73\20	0.88\15	0.87\22	0.99\15
RA	0.84\1	0.86\19	0.92\13	0.92\11	0.93\3	0.77\12	0.90\11	0.89\17	1.00\1
AA	0.83\4	0.87\10	0.88\20	0.91\14	0.93\3	0.72\22	0.90\11	0.88\19	0.99\15
JC	0.82\17	0.88\5	0.85\21	0.90\18	0.91\16	0.66\26	0.84\22	0.79\26	0.95\24
PA	0.72\25	0.79\24	0.81\25	0.59\27	0.88\23	0.65\27	0.83\25	0.88\19	0.90\26
CAR	0.81\20	0.87\10	0.90\16	0.91\14	0.90\20	0.73\20	0.86\19	0.91\13	0.99\15
CRA	0.82\17	0.89\1	0.93\9	0.91\14	0.93\3	0.77\12	0.89\14	0.92\11	1.00\1
CAA	0.83\4	0.85\21	0.94\2	0.92\11	0.91\16	0.76\14	0.88\15	0.91\13	1.00\1
CJC	0.83\4	0.87\10	0.90\16	0.91\14	0.91\16	0.76\14	0.86\19	0.88\19	0.99\15
CPA	0.82\17	0.87\10	0.90\16	0.90\18	0.90\20	0.74\18	0.87\18	0.92\11	0.97\21
NMF	0.70\26	0.76\25	0.85\21	0.86\22	0.89\22	0.69\24	0.86\19	0.89\17	0.99\15
Cos	0.80\23	0.84\22	0.84\24	0.82\24	0.87\24	0.76\14	0.84\22	0.83\24	0.96\23
Euc	0.73\24	0.69\27	0.68\27	0.60\26	0.79\27	0.67\25	0.76\26	0.80\25	0.87\27
Jac	0.81\20	0.84\22	0.85\21	0.84\23	0.86\25	0.76\14	0.84\22	0.87\22	0.97\21
Pea	0.68\27	0.70\26	0.69\26	0.62\25	0.82\26	0.70\23	0.75\27	0.77\27	0.91\25
BPR	0.84\1	0.89\1	0.92\13	0.90\18	0.92\11	0.74\18	0.88\15	0.91\13	0.99\15
NBI	0.83\4	0.87\10	0.90\16	0.92\11	0.91\16	0.78\11	0.91\6	0.91\13	1.00\1
SRNMF-CN	0.84\1	0.88\5	0.94\2	0.95\1	0.93\3	0.83\2	0.91\6	0.93\9	1.00\1
SRNMF-RA	0.83\4	0.88\5	0.93\9	0.94\5	0.92\11	0.82\3	0.91\6	0.94\3	1.00\1
SRNMF-AA	0.83\4	0.88\5	0.94\2	0.94\5	0.93\3	0.82\3	0.91\11	0.93\9	1.00\1
SRNMF-JC	0.83\4	0.88\5	0.93\9	0.94\5	0.92\11	0.85\1	0.91\6	0.94\3	1.00\1
SRNMF-PA	0.83\4	0.87\10	0.94\2	0.95\1	0.93\3	0.82\3	0.92\1	0.94\3	1.00\1
SRNMF-CAR	0.83\4	0.89\1	0.93\9	0.95\1	0.93\3	0.81\9	0.91\6	0.94\3	1.00\1
SRNMF-CRA	0.83\4	0.87\10	0.94\2	0.94\5	0.94\1	0.82\3	0.92\1	0.94\3	1.00\1
SRNMF-CAA	0.83\4	0.87\10	0.94\2	0.94\5	0.92\11	0.82\3	0.92\1	0.95\2	1.00\1
SRNMF-CJC	0.83\4	0.87\10	0.95\1	0.94\5	0.93\3	0.80\10	0.92\1	0.96\1	1.00\1
SRNMF-CPA	0.83\4	0.89\1	0.94\2	0.95\1	0.94\1	0.82\3	0.92\1	0.94\3	1.00\1

We compare our SRNMF method with seventeen well-known methods presented in baseline algorithms. For each real network, 10% of its links will be randomly selected to constitute the probe set, and the rest of the links constitute the training set. Prediction accuracy is measured by AUC. The numbers behind the slash denote the ranking.

Table 2 shows the comparison of precision for nine real-world networks. Our SRNMF methods are in red color text while the baseline methods are in black color text. The numbers behind the slash denote the ranking. For example, 0.31\18 means the precision is 0.31, and the whole ranking in all methods is 18. This table shows that the proposed SRNMF (including SRNMF-CN, SRNMF-RA, SRNMF-AA, SRNMF-JC, SRNMF-PA, SRNMF-CAR, SRNMF-CRA, SRNMF-CAA, SRNMF-CJC and SRNMF-CPA) framework outperforms the LCP-based (including CAR, CRA, CAA, CJC and CPA), CN-based (including CN, RA, AA, JC, PA), Projection-based (including Jac, Euc, Cos, Pea, BPR, NBI) and NMF algorithms. Based on the results, we can draw conclusion that the LCP-based methods perform better than the CN-based methods and NMF methods. The reason is that the LCP-based methods additionally concerns the information derived from the node neighbourhood connectivity. NBI and BPR methods perform better than other projection-based methods. In addition, our proposed SRNMF framework performs better than LCP-based methods by adding similarity-based regularization. Moremore, SRNMF is superior to projection-based methods which cause loss of the original topological information in the bipartite network structure. Such as on Enzymes network, an improvement of 106% is offered in average precision compared to similarity-based methods, and an improvement of 125.6% is offered in average precision compared to projection-based methods. This finding provides a strong evidence that methods using manifold learning and similarity regularized are more robust than other baseline methods.

Moreover, Table 3 demonstrates again a clear superiority on AUC index. Based on the results, A conclusion is drawn that the LCP-based (including CAR, CRA, CAA, CJC and CPA) methods almostly perform better than the CN-based (including CN, RA, AA, JC, PA), Projection-based (including Jac, Euc, Cos, Pea, BPR, NBI) and NMF methods. And our proposed SRNMF algorithms perform the best. Such as on SW network, an improvement of 12.6% is offered in average AUC compared with similarity-based methods, and an improvement of 11.7% is offered in average AUC compared with projection-based methods. Besides, our SRNMF methods perform better than benchmark methods (text in black color) in terms of stabilty.

**Figure 2:**
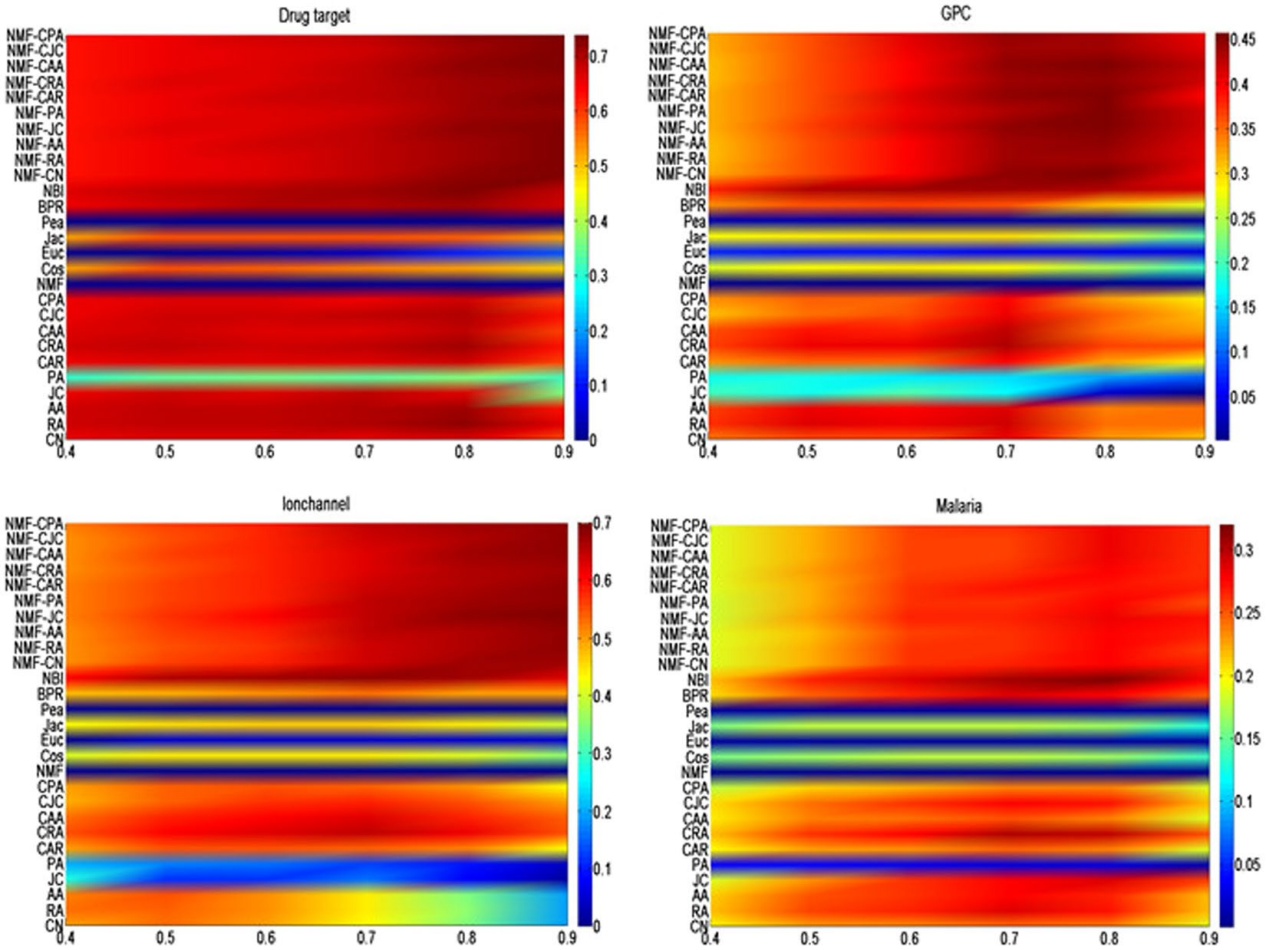
Precision under different methods with different sizes of training sets on four real networks. We compare our SRNMF method (including SRNMF-CN, SRNMF-RA, SRNMF-AA, SRNMF-JC, SRNMF-PA, SRNMF-CAR, SRNMF-CRA, SRNMF-CAA, SRNMF-CJC and SRNMF-CPA) with seventeen well-knowm methods (including CN, RA, AA, JC, PA, CAR, CRA, CAA, CJC,CPA, NMF, Cos, Euc, Jac, Pea, BPR and NBI) presented in baseline algorithms and the precision is returned with the average over 100 runs. X-axis denotes the fraction of links in trainging set. Y-axis denotes the each method.

**Figure 3:**
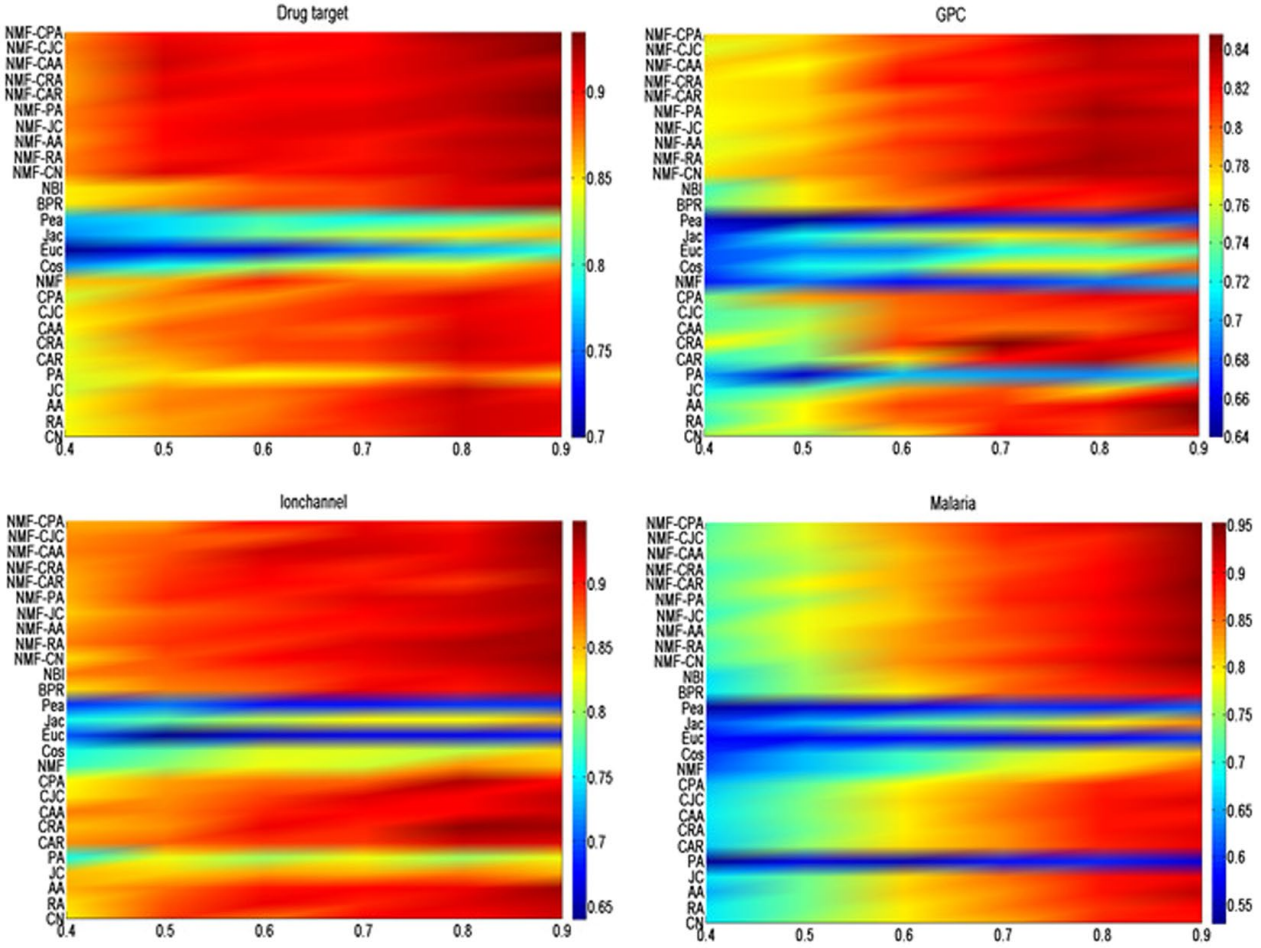
AUC under different methods with different sizes of training sets on four real networks. We compare our SRNMF method (including SRNMF-CN, SRNMF-RA, SRNMF-AA, SRNMF-JC, SRNMF-PA, SRNMF-CAR, SRNMF-CRA, SRNMF-CAA, SRNMF-CJC and SRNMF-CPA) with seventeen well-known methods (including CN, RA, AA, JC, PA, CAR, CRA, CAA, CJC, CPA, NMF, Cos, Euc, Jac, Pea, BPR and NBI) presented in baseline algorithms and the AUC is returned with the average over 100 runs. X-axis denotes the fraction of links in trainging set. Y-axis denotes the each method.

Experiments under different fractions (from 40% to 90%) of four datasets (drug target, GPC, Ionchannel, malaria datasets) are conducted to test the accuracies for link prediction in bipartite networks. Results are shown in Figs 2 and 3 respectively. Each value of the accuracy is returned with the average over 100 runs with independently random network divisions of the training set and probe set. The number of predicted links, L, is always set as being equal to the size of the probe set. According to Figs 2 and 3, by varying the size of training set, prediction accuracies of SRNMF (including SRNMF-CN, SRNMF-RA, SRNMF-AA, SRNMF-JC, SRNMF-PA, SRNMF-CAR, SRNMF-CRA, SRNMF-CAA, SRNMF-CJC and SRNMF-CPA) methods are either the best or very close to the best, other benchmark algorithms (especially PA, NMF and Pea) give very poor predictions for some networks. Usually, larger training set contains more information which could make the prediction easier. However, as shown in Figs 2 and 3, the precision and AUC do not always increase with the size of training set.

**Figure 4:**
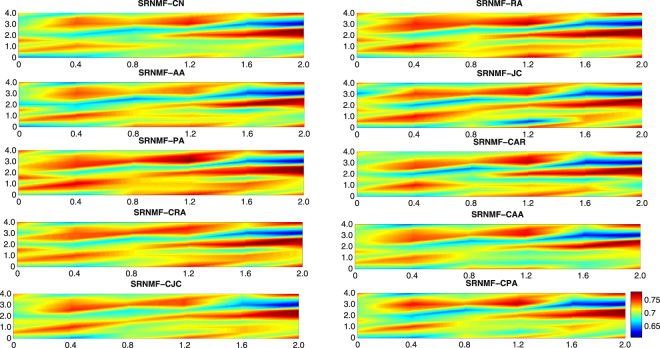
Precision sensitivity analysis on drug-target dataset. The precision results of our proposed SRNMF framework (including SRNMF-CN, SRNMF-RA, SRNMF-AA, SRNMF-JC, SRNMF-PA, SRNMF-CAR, SRNMF-CRA, SRNMF-CAA, SRNMF-CJC and SRNMF-CPA) using the drug-target dataset. X-axis denotes *γ* value, Y-axis denotes *λ* value.

As we know, the choice of parameters influences evaluation results. Our SRNMF model has two regularization parameters *γ* and *λ*. To show how the precision preformance of SRNMF varies with the parameters *γ* and *λ*, we choose drug-target network as an example in this paper and the results are depicted in Fig. 4. As seen from Fig. 4, SRNMF achieves consistently good performance when *λ* varies from 1.5 to 2.5 and *γ* varies from 1.5 to 2 with the different similarity measures.

## Discussion

In this paper, we investigate the problems of link prediction in bipartite network and propose a framework based on similarity-based regularized latent feature model (SRNMF), which exploits the intrinsic topological structure of the nodes and encodes the geometrical information of the networks by constructing a similarity-based matrix. By preserving the similarity structure, our framework is more powerful in discrimination than the latent feature model. The new framework takes advantages of latent feature model and topological structure. A unified object function framework is proposed to derive the SRNMF in terms of NMF loss function and similarity-based regularization. The SRNMF can be optimized by applying the method of gradient descent. The results demonstrate a more effective, robust and stabilized performance of our SRNMF framework compared with the state-of art methods.

We compare the proposed SRNMF framework with other seventeen baseline methods on nine real-world datasets. These methods can be classified into bipartite-based methods and projection-based methods. Bipartite-based methods directly predict links in the bipartite network, and projection-based methods project the bipartite network into two monopartite networks to predict new links. Cos, Euc, Jac, Pea, BPR, NBI belong to projection-based methods. The rest baseline methods and our SRNMF methods are bipartite-based methods. In general, bipartite-based methods performs better than projection-based methods, because projection-based methods cause loss of the original topological information in the bipartite network structure. By adding similarity-based regularization, our SRNMF methods are siginificantly superior to other bipartite-based methods in terms of accuracy and stablity. Despite the passable performance of projection-based methods, NBI and BPR exhibit the higher AUC and prediction values.

Some extensions of this work can be explored. One of the concerns is the drawback of NMF, since its high complexity of iterative calculation. To reduce the computational complexity, parallelization^37,38^ and sampling methods can be adopted. Also more efficient optimization algorithms can be reconsidered to obtain the global optimal solution in NMF. Moreover, the weight to improve the link prediction accuracy in a bipartite network has not been reasearched systematically, which is important to be explored in the future.

## Methods

### Similarity-base Regularized Nonnegative Matrix Factorization (SRNMF)

NMF obtains parts-based representation due to the nonnegative constraints. However, the intrinsic geometrical and discriminating structure of the node space cannot be revealed. In this section, we introduce our SRNMF algorithm by incorporating a similarity based regularizer, which avoids the limitation.

#### Determination of the number of latent features

There are many methods to determine the number of latent features, such as Partition density, Bayesian information criterion and cross validation. These methods need to calculate each possible value of the latent features under each number. Thus they are too complex in computation to be used in real networks. The PCA^39^ is used to reduce the dimensionality of a matrix consisting of a large number of interrelated variables, while still retaining the maximum information of the variation present in the matrix. This is achieved by transforming the original matrix to a new set of variables, named principal components (PCs), which are uncorrelated and ordered with the first few components explaining most of the variation present in all of the original variables. The eigenvalues of the matrix are used to calculate the cumulative contribution rate to determine the number of dimension. So in this paper, we determine the number of latent features by calculate the cumulative contribution rate and cumulative contribution rate of 95% is adopted to choose PCs.

#### NMF with Manifold Regularization

NMF aims to find two nonnegative matrices whose product provides a good approximation to the original matrix. A natural assumption here could be that if two nodes *u*_*i*_, *v*_*j*_ are close in the intrinsic geometry of the node distribution, then *A*_*ij*_ and (*XY*)_*ij*_ are also close to each other. *A*_*ij*_ and (*XY*)_*ij*_ are the connected representations of these two nodes from the original network and a low-dimensional approximation derived from NMF. This assumption is so-called local invariance assumption^40,41^. It has been shown that learning performance can be significantly enhanced if the topological similarity structure is exploited and the local invariance is considered.

*S*_*ij*_ is used to measure the closeness of two nodes *u*_*i*_ and *v*_*j*_. The different similarity measures such as CN, AA, RA, JC, PA, CAR, CRA, CAA, CJC, CPA can be used in this paper (for details see baseline algorithms). With the above defined similarity matrix *S*, we can use the following term to measure the smoothness of the low dimensional representation

16 $$R=\frac{{\rm{1}}}{{\rm{2}}}\sum _{i=1}^{n}\sum _{j=1}^{m}{({A}_{ij}-\sum _{k=1}^{K}{x}_{ik}\cdot {y}_{kj})}^{2}\cdot {S}_{ij}$$

By miniming *R*, we expect that if two nodes *u*_*i*_ and *v*_*j*_ are close (i.e., *S*_*ij*_ is big), *A*_*ij*_ and ${\sum }_{k=1}^{K}{x}_{ik}\cdot {y}_{kj}$ are also close to each other. Combing this similarity-based regularizer with the original NMF objective function leads to our SRNMF. We now consider Euclidean distance formulations of NMF latent feature as the optimization problem, so the proposed model can be defined as the following constrained nonlinear programming

17 $$\begin{array}{llll}{\rm{\min }} & {O}_{{1}}(x,y) & = & \frac{{\rm{1}}}{{\rm{2}}}{\Vert A-XY\Vert }_{F}^{2}+\gamma R+\lambda {\Vert XY\Vert }_{\ast }\\ s\mathrm{.}t\mathrm{.} &  &  & X\ge \mathrm{0,}\\  &  &  & Y\ge 0\end{array}$$

Here, *λ* ≥ 0 and γ ≥ 0 are the balance parameters, ||*XY*||_*_ is the nuclear norm which is the sum of the singular values of *XY*. The benefit of the nuclear norm regularization is that, with a sufficiently large regularization parameter, the final solution will be low-rank^42^.

We utilize a standard reformulation of the nuclear norm which is more flexible to manipulate^43^.

18 $${\Vert XY\Vert }_{\ast }=\frac{{\rm{1}}}{{\rm{2}}}\mathop{min}\limits_{X,Y}({\Vert X\Vert }_{F}^{2}+{\Vert Y\Vert }_{F}^{2})$$

Combining Equation (17) and Equation (18), the objective function of our proposed SRNMF model can be rewritten as

19 $$\begin{array}{llll}{\rm{\min }} & O(x,y) & = & \frac{1}{2}\sum _{i=1}^{n}\sum _{j=1}^{m}{({A}_{ij}-\sum _{k=1}^{K}{x}_{ik}\cdot {y}_{kj})}^{2}+\frac{1}{2}\gamma \sum _{i=1}^{n}\sum _{j=1}^{m}{({A}_{ij}-\sum _{k\mathrm{=1}}^{K}{x}_{ik}\cdot {y}_{kj})}^{2}\cdot {S}_{ij}\\  &  &  & +\,\frac{1}{2}\lambda (\sum _{i}\sum _{p}{x}_{ip}^{2})+\frac{1}{2}\lambda (\sum _{j}\sum _{q}{y}_{qj}^{2})\\ s\mathrm{.}t\mathrm{.} &  &  & {x}_{ik}\ge \mathrm{0,}\\  &  &  & {y}_{kj}\ge 0\end{array}$$

The objective function *O*(*x*, *y*) in (19) is not convex in both *x* and *y* together. Therefore, it is unrealistic to expect an algorithm to find the global minima. To address this problem, two iterative algorithms are introduced.

Let *φ*_*ik*_ and *ψ*_*kj*_ be the lagrange multipliers for constraint *x*_*ik*_ ≥ 0 and *y*_*kj*_ ≥ 0, respectively. The Lagrange *L* is:

20 $$\begin{array}{rcl}L & = & \frac{1}{2}\sum _{i=1}^{n}\sum _{j=1}^{m}{({A}_{ij}-\sum _{k=1}^{K}{x}_{ik}\cdot {y}_{kj})}^{2}+\frac{1}{2}\gamma \sum _{i=1}^{n}\sum _{j=1}^{m}{({A}_{ij}-\sum _{k=1}^{K}{x}_{ik}\cdot {y}_{kj})}^{2}\cdot {S}_{ij}\\  &  & +\frac{1}{2}\lambda (\sum _{i}\sum _{p}{x}_{ip}^{2})+\frac{1}{2}\lambda (\sum _{j}\sum _{q}{y}_{qj}^{2})+\sum _{i}\sum _{k}{{\phi }}_{ik}{x}_{ik}+\sum _{k}\sum _{j}{\psi }_{kj}{y}_{kj}\end{array}$$

The partial derivatives of *L* with respect to *x*_*ik*_ and *y*_*kj*_ are

21 $$\begin{array}{ll}\frac{\partial L}{\partial {x}_{ik}} & =-\sum _{j}\mathrm{[(1}+\gamma {S}_{ij})\cdot {A}_{ij}\cdot {y}_{kj}]+\sum _{j}\mathrm{[(1}+\gamma {S}_{ij})\cdot (\sum _{k}{x}_{ik}\cdot {y}_{kj})\cdot {y}_{kj}]+\lambda {x}_{ik}+{{\phi }}_{ik}\\  & =-{(A{Y}^{T})}_{ik}-\gamma {[(S\cdot A){Y}^{T}]}_{ik}+{[(XY){Y}^{T}]}_{ik}+\gamma {[S\cdot (XY){Y}^{T}]}_{ik}+\lambda {X}_{ik}+{{\phi }}_{ik}\end{array}$$

22 $$\begin{array}{ll}\frac{\partial L}{\partial {y}_{kj}} & =-\sum _{i}\mathrm{[(1}+\gamma {S}_{ij})\cdot {A}_{ij}\cdot {x}_{ik}]+\sum _{i}\mathrm{[(1}+\gamma {S}_{ij})\cdot (\sum _{k}{x}_{ik}\cdot {y}_{kj})\cdot {x}_{ik}]+\lambda {y}_{kj}+{\psi }_{kj}\\  & =-{({X}^{T}A)}_{kj}-\gamma {[{X}^{T}(S\cdot A)]}_{kj}+{({X}^{T}XY)}_{kj}+\gamma {[{X}^{T}[S\cdot (XY)]]}_{kj}+\lambda {Y}_{kj}+{\psi }_{kj}\end{array}$$

Using the KKT conditions *φ*_ik_ *x*_*ik*_ = 0 and ${\psi }_{kj}{y}_{kj}=0$, we get the following equations for *x*_*ik*_ and *y*_*kj*_

23 $$\begin{array}{l}-{(A{Y}^{T})}_{ik}{x}_{ik}-\gamma {[(S\cdot A){Y}^{T}]}_{ik}{x}_{ik}+{[(XY){Y}^{T}]}_{ik}{x}_{ik}+\gamma {[S\cdot (XY){Y}^{T}]}_{ik}{x}_{ik}+\lambda {X}_{ik}{x}_{ik}=0\end{array}$$

24 $$\begin{array}{l}-{({X}^{T}A)}_{kj}{y}_{kj}-\gamma {[{X}^{T}(S\cdot A)]}_{kj}{y}_{kj}+{({X}^{T}XY)}_{kj}{y}_{kj}+\gamma {[{X}^{T}[S\cdot (XY)]]}_{kj}{y}_{kj}+\lambda {Y}_{kj}{y}_{kj}=0\end{array}$$

These equations lead to the following updating rules:

25 $${x}_{ik}\leftarrow {x}_{ik}\cdot \frac{{[A{Y}^{T}+\gamma (S\cdot A){Y}^{T}]}_{ik}}{{[XY{Y}^{T}+\gamma [S\cdot (XY)]{Y}^{T}+\lambda X]}_{ik}}$$

26 $${y}_{kj}\leftarrow {y}_{kj}\cdot \frac{{[{X}^{T}A+\gamma {X}^{T}(S\cdot A)]}_{kj}}{{[{X}^{T}XY+\gamma {X}^{T}[S\cdot (XY)]+\lambda Y]}_{kj}}$$

**Algorithm 1:**
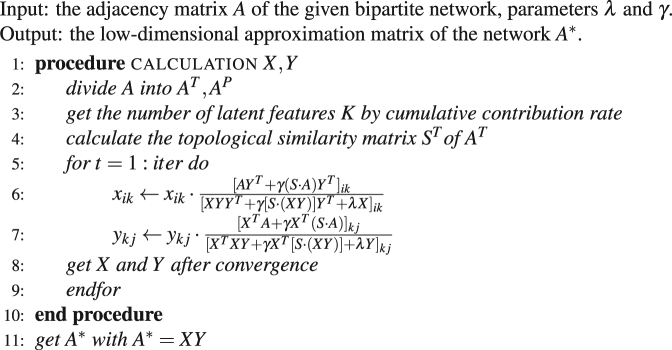
The proposed SRNMF framework.

### The proposed SRNMF framework

The low-dimensional approximation matrix of the network *A* ^*^ can be obtained by the above optimal procedures and the pseudocode is presented in algorithm 1.

### Complexity analysis

Here, we give a simple complexity analysis of the proposed SRNMF framework. The most time-consuming part occurs in updating *X* and *Y*. For each iteration, the time cost of $$(A{Y}^{T}+\gamma (S\cdot A){Y}^{T})$$ is $$O(|V||W|K+|V||W|K+|V||W|)$$, the time cost of $$(XY{Y}^{T}+\gamma (S\cdot (XY)){Y}^{T}+\lambda X)$$ is $$O(|V|{K}^{2}+|W|{K}^{2}+$$ $$|V||W|K+|V||W|+|V|K)$$, thus the total time cost of the algorithm is $$O({N}_{iter}(|V||W|K+|V|{K}^{2}+|W|{K}^{2}+$$ $$|V||W|+|V|K)) \sim O({N}_{iter}(|V||W|K))$$, where *N*_*iter*_ is the number of iterations, $$|V|$$ and $$|W|$$ denote the number of two different types of nodes respectively. Many real-world networks are known to be sparse, so the final time cost can be denoted as $$O({N}_{iter}(|E|K))$$, where $$|E|$$ is the number of the edges in the bipartite network.
